# Isolation and characterization of a thermophilic *Bacillus* sp. with protease activity isolated from hot spring of Tarabalo, Odisha, India

**Published:** 2013-06

**Authors:** Mrunmaya Kumar Panda, Mahesh Kumar Sahu, Kumananda Tayung

**Affiliations:** 1 Department of Bioinformatics, North Orissa University, Takatpur, Baripada-757003, Odisha, India; 2 Department of Botany, North Orissa University, Takatpur, Baripada-757003, Odisha, India

**Keywords:** Thermophilic *Bacillus*, Protease, Phylogenetic analysis, G+C content

## Abstract

**Background and Objectives:**

Thermophilic bacteria are less studied but important group of microorganisms due to their ability to produce industrial enzymes.

**Materials and Methods:**

In this study, thermophilic bacteria were isolated from hot spring of Tarabalo, India. A bacterium that could tolerate high temperatures was characterized by morphology, biochemistry and sequencing of its 16S rRNA gene. The isolate was screened for protease and amylase activity. Phylogenetic affiliations and G+C content of the isolate was studied.

**Results:**

The bacterium with the ability to tolerate high temperatures was identified as *Bacillus* sp. both by morphology, biochemistry and sequencing of its 16S rRNA gene. BLAST search analysis of the sequence showed maximum identity with *Bacillus amyloliquefaciens* (99% similarity). Strain exhibited considerable protease activity. Phylogenetic analysis of the isolate revealed close affiliation with thermophilic *Bacillus* species. The G+C content was found to be 54.7%.

**Conclusion:**

The study confirmed that the isolated *Bacillus* sp. to be a true thermophile and could be a source of thermostable protease which can be exploited for pharmaceutical and industrials applications.

## INTRODUCTION

Bacteria are ubiquitous and highly diverse. They can survive in all sorts of inhospitable environments. Studies in the last two decades have revealed that 99% of bacteria present in the environment are still unexplored or overlooked in laboratory cultivation and hence remain obscure for their ecological functions and unexploited for biotechnological applications (1). Thermophilic bacteria are microbes that mostly inhabit hot springs, live and survive in temperatures above 70°C. They have been less explored due to difficulties in isolation and maintenance of pure culture. Therefore, their diversity and biotechnological potential remains to explored from majority of the thermal habitats. As a consequence of growth at high temperatures and unique macromolecular properties, thermophiles can possess high metabolism, physically and chemically stable enzymes and lower growth but higher end product yields than similar mesophilic species (2).

Thermophilic microorganisms have gained world-wide importance due to their tremendous potential to produce thermostable enzymes that have wide applications in pharmaceuticals and industries (3). Proteases are such enzymes which account for nearly 60% of the total world-wide enzyme sales (4). Thermostable proteases are of greater advantage in applications because they not only do not usually denature at high temperatures, but they also remain active at such temperatures. Several workers have reported thermophilic bacteria from diverse environmental habitats such as geothermal sites and hot springs. The state of Odisha, situated in eastern region of India, is also rich in hot springs. One such hot spring is Tarabalo. The microbial diversity of this hot spring has not yet been fully studied, but there are few sporadic reports by Rath *et al*. (5). Therefore, the aims of this study were to isolate thermophilic bacteria from muddy soil samples of Tarabalo hot springs, determine the thermostability of the isolates, screen for industrial enzymes and study the phylogenetic affiliation of the thermophilic bacterium in comparison with other bacterial isolates occurring as mesophiles, thermophiles and hyperthermophiles.

## MATERIALS AND METHODS

### Study site & collection of samples

The hot spring Tarabalo is situated at the district of Nayagarh, Odisha, India. It is the second largest hot spring of India, as per the report of Tourism Development Corporation India. Tarabalo hot spring is situated at 20° 14’ 49.38” E Longitude and 85° 19’ 11.04”N Latitude. Before exploration the place was a paddy field. Due to its remoteness it is less influence by human interferences and believed to have rich microbial wealth. The temperature of the hot spring during the sampling period was about 60°C. The pH was recorded to be in the range of 8-9 indicating alkaline environment. Samples (muddy soil) were collected from different sites of the hot spring in sterile poly bags and immediately brought into the laboratory.

### Isolation of bacteria

Bacteria were isolated in Nutrient Agar (NA) medium (Himedia, Mumbai) India following serial dilution technique. The procedure adopted was as follows: 10 gram of soil sample was diluted in 90 ml of sterile distilled water in 250 ml conical flask and kept it a orbital shaker at 150 rpm to get a homogenized soil suspension. Serial dilution was made and dilution of 10^−7^ was inoculated into NA plates and incubated at 37°C for 24 h. Isolated colonies growing on each diluted plates were transferred into freshly prepared nutrient agar slants. The bacterial strains isolated on NA slants were kept at in refrigerator for further study.

### Determination for thermo-tolerance

Pure cultures of the bacterial isolates were determined for their thermophilic characteristics. Each bacterial isolates were inoculated into 5 ml of nutrient broth medium in test tubes. The tubes were incubated at initial temperature of 50°C for 12 h. After specified incubation period each broth culture of bacteria were streaked onto freshly prepared Nutrient Agar medium. Bacterial isolates growing in the plates were selected and again tested for their thermo-tolerance at higher temperature. Finally a bacterium that could tolerate temperature of 90°C was selected for further study.

### Identification and characterization of the isolate

The selected strain was observed morphologically and growth characteristics were studied. The isolate was characterized by Gram staining technique. Based on Gram's staining the strain was found to be Gram-positive and microscopic observation revealed rod shaped bacterium arranged in chain. Various biochemical tests like endospore formation, motility, anaerobic; catalase and oxidase tests were performed. Morphological, microscopic observation and biochemical test indicated the bacterium to be *Bacillus* sp.

### Enzyme assay

The strain was determined for production of enzymatic activity if any. Two tests were conducted i.e. test for protease and amylase activity. For protease activity, Skimmed Milk Agar (SMA) medium was prepared and the nutrient broth culture of bacterium after 24 h of incubation was spot inoculated following agar well method. After inoculation the SMA plate was incubated at 37°C for 24-48 h. The plate was observed for halo zone around the well. Observation of halo zone indicated positive protease activity. Similarly, amylase activity was determined in starch agar (SA) medium. Starch agar plates were prepared and spot inoculated with the bacterial strain. After incubating the plates at 37°C for 24-48 h, the plates were observed for clear zone near inoculated colony after treating with Iodine vapour.

### PCR amplification and 16Sr DNA Sequencing

Genomic DNA was extracted and purified according to Sambrook and Russell (6) and its purity was spectrophotometrically assessed by the A260/A280 ratio. Sequencing of 16S rDNA of the isolate and amplification of the target gene was done using Big Dye Chemistry, and performed as per the manufacturer's protocols (Applied Biosystems, USA). Universal bacterial primer 1492R (5’- TAC GGY TAC CTT GTT ACG ACT T-3’) and the domain bacteria-specific primer 27F (5’- AGA GTT TGA TCM TGG CTC AG-3’) were used for 16S rDNA amplification (7). The PCR product was purified using QIA quick PCR purification kit (Qiagen). Purified 16S rDNA was sequenced partially using ABI PRISM big dye terminator cycle sequence reading reaction kit (Applied Biosystems) under the following conditions: initial denaturation at 94°C for 5 min; 30 cycles of denaturation at 94° for 45 sec, annealing at 48°C for 45 sec, extension at 72°C for 90 sec, and a final extension at 72°C for 5 min. The purified sequencing reaction mixtures were electophoresed using an Applied Biosystems model 310 automatic DNA sequencer (Perkin Elmer, Massachusetts USA). The sequence was annotated and submitted to GenBank.

### Homology search and phylogenetic analysis

The 16S rDNA sequence of the isolate was been compared with the non redundant databases in all GenBank + EMBL + DDBJ + PDB sequences (but no EST, STS, GSS, environmental samples or phase 0, 1 or 2 HTGS sequences) using the program BLASTN 2.2.25 by selecting the optimization parameter to highly similar sequences (Megablast). Phylogenetic study was conducted by considering 16S rDNA sequences of 30 bacterial isolates belonging to mesophiles, thermophiles and hyperthermophiles. The tree was generated by Minimum Evolution (ME) method using MEGA 4.0 (8).

### GC% study

Guanine plus cytosine percentage was studied in 16 isolates belonging to three bacterial groups (i.e. mesophiles, thermophiles and hyper- thermophiles). It was calculated using the software ACUA (9). The sequences of all bacterial isolates were selected including sequence of own isolate and uploaded in input files. The GC% option was selected and the program was run and result was obtained.

## RESULTS

Altogether 22 bacterial isolates were obtained from muddy soil samples collected from hot spring of Tarabalo Odisha, India. The bacterial isolates were screened for their thermo-tolerance property in different temperatures starting from 50°C to 90°C. Out of the total isolates only one strain could sustain temperatures of 90°C. The bacterium was characterized by morphological observation and biochemical tests. Gram staining, microscopic and biochemical characterization revealed it to be a Gram positive rod.

**Fig. 1:**
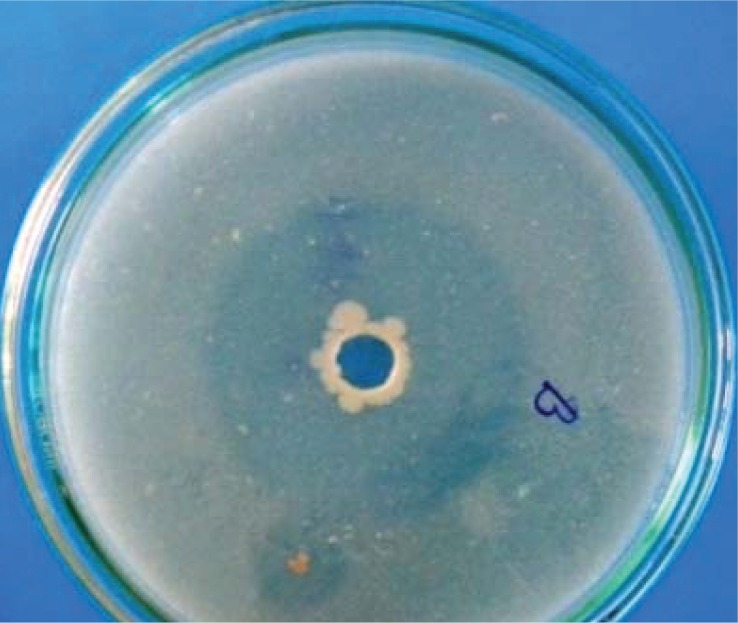
Protease activity.

The bacterium tested positive for motility, catalase activity, endospore production and negative test from anaerobic and sulphate reduction. The biochemical characteristic of the bacterium is presented in Table 1. Morphological and biochemical characterization of the isolate indicated it to be *Bacillus* sp. Species level confirmation of the isolate was done by 16S rDNA sequencing. Based on BLAST search analysis of the sequence, the isolate showed maximum identity with *Bacillus amyloliquefaciens* (99%) having accession number CP002627.1. The sequence of the isolate has been deposited in GenBank with accession number JQ962977. The isolate was screened for both protease and amylase activity. The bacterium showed considerable protease activity (Fig. 1) but no amylase activity was observed.

**Table 1:** Biochemical characteristics of thermophilic *Bacillus* sp.

CHARACTERSTICS	Observation
Rod shaped in young cultures	+
Diameter over 2.5 mM	−
Filaments	−
Rods or filaments curved	−
Cocci in tetrads or packets	−
Endospores Produced	+
Motile	+
Stain Gram positive at least in young cultures	+
Strict aerobes	ND
Facultative anaerobes or microaerophiles	ND
Strict anaerobes	−
Product of carbohydrate fermentation	ND
Sulfate actively reduced to sulfide	−
Catalase	+
Oxidase	ND
Marked acidity from glucose	+
Nitrate reduced to nitrite	ND
Requires 3-12% NaCl for growth	ND
Ability to grow at >700 C	+

+ indicates positive and – indicates negative test; ND- not determined

**Fig. 2:**
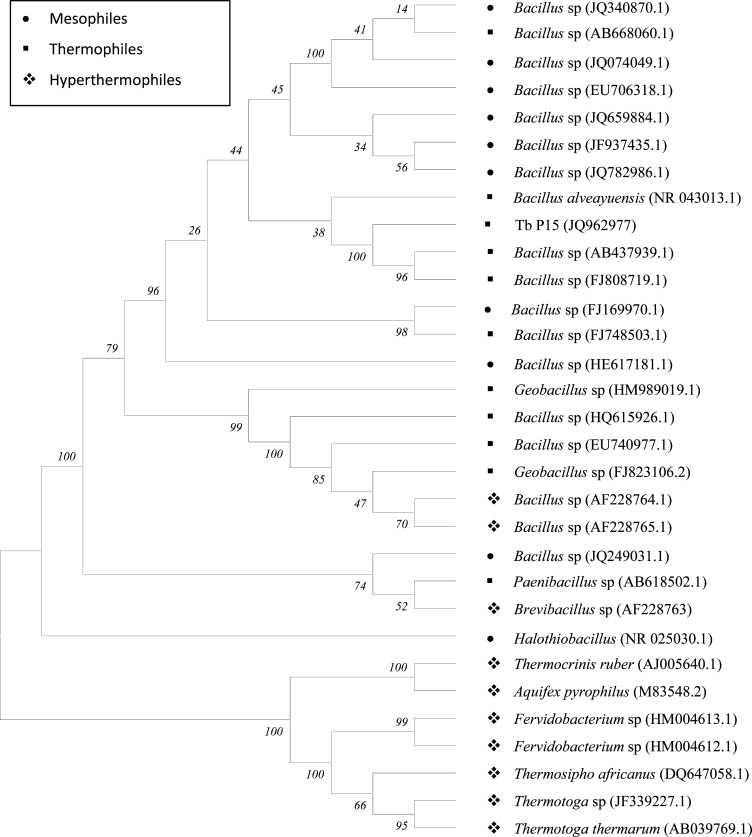
Phylogenetic tree generated by Minimum Evolution method of 31 taxa representing mesophilic, thermophilic and hyperthermophilic bacteria. The optimal tree with the sum of branch length = 0.85161164 is shown. The percentage of replicate trees in which the associated taxa clustered together in the bootstrap test are shown next to the branches. The evolutionary distances were computed using the Maximum Composite Likelihood method and are in the units of the number of base substitutions per site. Tb P15 is own isolate. The values in parenthesis indicate accession no. of the isolates.

**Fig. 3:**
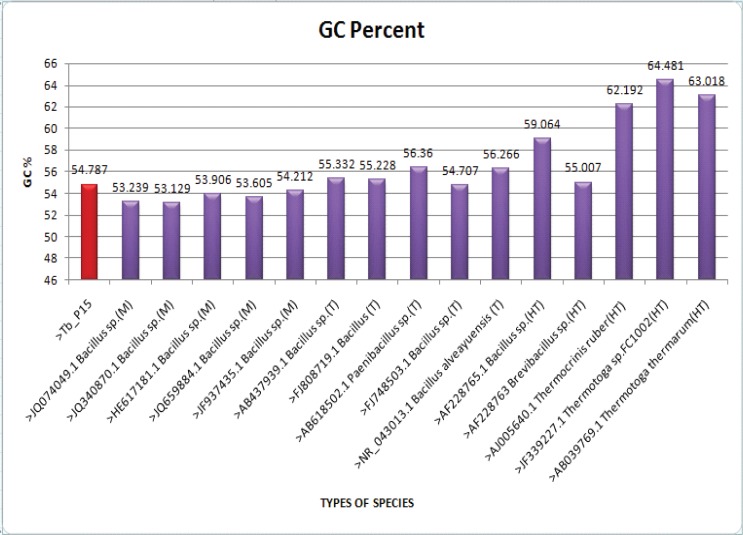
GC percentage of the *Bacillus* isolates along with other bacterial isolates occurring as mesophiles (M), thermophiles (T) and hyperthermophiles (HT). The red bar indicates GC content of own isolate.

Molecular phylogeny of the isolate was analysed considering 30 bacterial taxa occurring as mesophiles, thermophiles and hyperthermophiles (Table 2). The generated tree revealed that the isolate shared close affinity with thermophilic *Bacillus* spp. having accession numbers AB437939.1 and FJ808719.1 respectively with well supported bootstrap value of 100% (Fig. 2). The tree also showed that hyperthermopiles forms a separate group with distinct clade supported by bootstrap of 100%. However, some of the hyperthermophiles also shared the same clade with thermophiles and mesophiles. The isolate was also studied for its GC content by comparing with 15 bacterial isolates (selected randomly) occurring as mesophiles, thermophiles and hyperthermophiles. The result indicated that the GC content of the isolate was 54.7% which is slightly higher than mesophiles but lower than the hyperthermophiles (Fig. 3).

**Table 2:** List of bacterial isolates with their isolation source, environment, country and accession numbers considered for the present study.

Bacteria	Accession no	Country	source	Environment
*Bacillus* sp.	FJ169970.1	China	Sea sediment	Mesophilic
*Bacillus* sp.	JQ074049.1	India	plant	Mesophilic
*Bacillus* sp.	EU706318.1	Kuwait	Coast of Kuwait	Mesophilic
*Bacillus* sp.	JF937435.1	China	Soil	Mesophilic
*Halothiobacillus* sp.	NR_025030.1	--	Hydrothermal	Mesophilic
*Bacillus* sp.	JQ249031.1	India	Soil	Mesophilic
*Bacillus* sp.	JQ340870.1	Iran	oil contaminated soil	Mesophilic
*Bacillus* sp.	HE617181.1	India	soda lake sediment	Mesophilic
*Bacillus* sp.	JQ659884.1	Singapore	plant tissue	Mesophilic
*Bacillus* sp.	JQ782986.1	China	marine sediment	Mesophilic
*Geobacillus* sp.	FJ823106.2	Korea	lakeshore duff	Thermophilic
*Bacillus* sp.	AB668060.1	Japan	hot spring	Thermophilic
*Bacillus* sp.	EU740977.1	China	hot spring	Thermophilic
*Bacillus* sp.	AB437939.1	Thiland	hot spring	Thermophilic
*Bacillus* sp.	FJ808719.1	Turkey	hot spring	Thermophilic
*Paenibacillus* sp.	AB618502.1	Japan	Fermented food	Thermophilic
*Geobacillus* sp.	HM989019.1	China	hot spring	Thermophilic
*Bacillus* sp.	FJ748503.1	Uganda	Landfill	Thermophilic
*Bacillus* sp.	HQ615926.1	China	soil	Thermophilic
*Bacillus alveayuensis*	NR_043013.1	--	deep-sea sediments	Thermophilic
*Bacillus* sp.	AF228764.1	Indonesia	LB media	Hyperthermophilic
*Bacillus* sp.	AF228765.1	Indonesia	LB media	Hyperthermophilic
*Brevibacillus* sp.	AF228763	Indonesia	oil wells	Hyperthermophilic
*Thermocrinis ruber*	AJ005640.1	USA	Yellow stone park	Hyperthermophilic
*Thermotoga* sp.	JF339227.1	Thailand	hot spring	Hyperthermophilic
*Thermotoga thermarum*	AB039769.1	Japan	oil reservoir	Hyperthermophilic
*Thermoshipo africa*nus	DQ647058	--	hot water	Hyperthermophilic
*Fervidobacterium* sp.	HM004613.1	China	hot spring	Hyperthermophilic
*Fervidobacterium* sp.	HM004612.1	China	hot spring	Hyperthermophilic
*Aquifex pyrophilus*	M83548.2	--	--	Hyperthermophilic

-- Not known

## DISCUSSION

Thermophilic microorganisms have the adaptability to survive in high environmental conditions. Many researchers believed that such capability may be due to their molecular modifications at cellular and subcellular level. In our present study muddy soil samples collected from hot spring of Tarabalo, Odisha, India was investigated for thermophilic bacteria. Out of the total isolates, one isolate was found to sustain temperatures up to 90°C. The ability of the bacterium to tolerate this temperature indicated to be thermophile. Narayan *et al*. (10) have also characterized bacterial isolates as thermophiles from Savusavu hotspring in Fiji by determining their growth at 90°C. The bacterium was identified as *Bacillus* sp. both by morphological and molecular characterization. Similar work carried out by Rath (11) has also reported thermophilic *Bacillus* and *Pseudomonas* species from three hot springs of Odisha, India. Thermophilic and hyperthermophilic microorganisms have the ability to produce wide variety of thermostable enzymes. Thermozymes are of great interest for industrial applications (12). Considering this facts, the thermophilic *Bacillus* isolate was screened for protease and amylase activity. Considerable protease activity was detected but no amylase activity was observed. Rath et al. (5) have also reported various extracellular enzymes from some thermophilic bacteria isolated from the same hot spring. However, those bacteria were not characterized. Several workers have reported protease activity from thermophilic *Bacillus* species (13, 14). Proteases are essential constituents of all forms of life on earth including prokaryotes, fungi, plants and animals. Proteases are highly exploited enzymes in food, leather, detergent, pharmaceutical, diagnostics, waste management and silver recovery (15). *Bacillus* protease is of special importance because of its wide applications in various industries like pharmaceutical, leather, food and waste processing industries (16). Phylogenetic study of the isolates with other bacterial isolates occurring as mesophiles, thermophiles and hyperthermophiles showed close affiliation with thermophilic *Bacillus* species and are grouped within the clade of thermophiles. This validates the isolate to be a thermophilic *Bacillus* species. The tree also revealed some hyperthermophiles shared same clade with thermophiles and mesophiles. Such ambiguity may be due biased in submission of sequences in Genbank. The GC content of the isolate was also found to be relatively high. This might be the reason for its ability to survive in high temperature. However, recent report of Wu *et al*. (17) suggests that higher GC content could not only be the sole reason for an organism to survive in extreme temperature. There were of the opinion that organisms isolated from soil preferably have higher GC content than aquatic isolates. Similarly, Hurst and Merchant (18) reported that high GC content is not an adaptation to high temperature among prokaryotes. Thus GC content cannot be taken into account to explain the adaptability of thermophiles in high temperature. Although this is not the first report of thermophilic *Bacillus* from hot spring of Tarabalo, but the organism is unique in the sense that it could survive temperatures of 90°C and could be a potential source of thermostable enzyme. Efforts are on to optimize the protease production in different temperature and pH and to purify the enzyme.
